# Preparation of Poly(glycidyl methacrylate) (PGMA) and Amine Modified PGMA Adsorbents for Purification of Glucosinolates from Cruciferous Plants

**DOI:** 10.3390/molecules25143286

**Published:** 2020-07-20

**Authors:** Li Cheng, Jianpeng Wu, Hao Liang, Qipeng Yuan

**Affiliations:** State Key Laboratory of Chemical Resource Engineering, Beijing University of Chemical Technology, Beijing 100029, China; buctchengli@163.com (L.C.); buctwujianpeng@163.com (J.W.)

**Keywords:** cruciferous plants, glucoerucin, PGMA, decolorization, purification

## Abstract

Glucosinolates (GLs) are of great interest for their potential as antioxidant and anticancer compounds. In this study, macroporous crosslinked copolymer adsorbents of poly (glycidyl methacrylate) (PGMA) and its amine (ethylenediamine, diethylamine, triethylamine)-modified derivatives were prepared and used to purify the GLS glucoerucin in a crude extract obtained from a cruciferous plant. These four adsorbents were evaluated by comparing their adsorption/desorption and decolorization performance for the purification of glucoerucin from crude plant extracts. According to the results, the strongly basic triethylamine modified PGMA (PGMA-III) adsorbent showed the best adsorption and desorption capacity of glucoerucin, and its adsorption data was a good fit to the Freundlich isotherm model and pseudo-second-order kinetics; the PGMA adsorbent gave the optimum decolorization performance. Furthermore, dynamic adsorption/desorption experiments were carried out to optimize the purification process. Two glass columns were serially connected and respectively wet-packed with PGMA and PGMA-III adsorbents so that glucoerucin could be decolorized and isolated from crude extracts in one process. Compared with KCl solution, aqueous ammonia was a preferable desorption solvent for the purification of glucoerucin and overcame the challenges of desalination efficiency, residual methanol and high operation costs. The results showed that after desorption with 10% aqueous ammonia, the purity of isolated glucoerucin was 74.39% with a recovery of 80.63%; after decolorization with PGMA adsorbent, the appearance of glucoerucin was improved and the purity increased by 11.30%. The process of using serially connected glass columns, wet-packed with PGMA and PGMA-III, may provide a simple, low-cost, and efficient method for the purification of GLs from cruciferous plants.

## 1. Introduction

Cruciferous vegetables are a rich source of glucosinolates (GLs); a large and diverse group of chemicals with cancer chemoprotective properties [1]. When plant cells are damaged, e.g., by grinding or chopping, GLs are released and converted into isothiocyanates by the enzyme myrosinase (thioglucoside glucohydrolase, EC3.2.3.1) [2]. Isothiocyanates are largely responsible for cancer chemoprevention and antioxidation [3,4,5]. Sulforaphane (4-methylsulfinybutylisothiocyanate), derived from glucoraphanin (4-methylsulfinybutylglucosinolate), is the most potent natural inducer of phase II (detoxification) enzymes, including quinone reductase and glutathione S-transferase [6] and has subsequently been shown to possess anticarcinogenic properties [7]. Erucin (4-methylthiobutyl isothiocyanate), derived from glucoerucin (4-methylsulfinylbutyl glucosinolate), has also shown promising anticancer effects in some in vitro and in vivo experiments [8,9]. Unlike sulforaphane, erucin and glucoerucin possess direct antioxidant behavior and are efficient scavengers of hydrogen peroxide and organic hydroperoxides. Therefore, we try to purify glucosinolate first and then produce isothiocyanate [10].

In the past few decades, GLs have been isolated and purified using solvent extraction and a variety of chromatographic techniques including: Alumina column chromatography [11]; low-pressure column chromatography [12]; preparative high-performance liquid chromatography [13]; preparative high-speed counter-current chromatography [14], ion-exchange chromatography on DEAE-Sephadex^®^ A-25 [10,15,16]; strong ion-exchange centrifugal partition chromatography [17]; and slow rotary counter-current chromatography [18]. However, the complexity and high operational costs of these methods are inappropriate for the large quantities of GLs necessary to satisfy the increasing demands of research and commerce. Therefore, an efficient and economical industrial approach to obtain the product should be developed to meet these demands.

GLs have good water solubility because of their ionized sulfate and hydrophilic β-D-thioglucose moieties [1]. Hence, macroporous ion-exchange resin adsorption [19] and alumina [12] have been used to isolate and concentrate GLs. Typically, an inorganic salt was used to desorb GLs from the ion-exchange resin; nanofiltration technology and precipitation with methanol were used to remove the salt from the isolated GLs. In a previous study, we found that precipitation with methanol could not remove inorganic salt completely [12]. Although nanofiltration technology could efficiently remove inorganic salt, it was not cost effective due to the specialized apparatus required. Therefore, it is necessary to develop an efficient and low-cost method to separate GLs without removing salt. Furthermore, large quantities of co-extracted pigments also compromised the purity of GLs. Until now, there have been few studies concerning the decolorization of the crude extracts of GLs from cruciferous vegetables.

Growing attention has been paid to separating and purifying pharmaceutical and natural products using polymeric resins because of their availability, economy, high chemically stability, and recyclability [20]. The aim of this study was to evaluate the performance (absorption/desorption and decolorization) of macroporous adsorbent PGMA and its three amine modified (ethylenediamine, diethylamine, triethylamine) anion-exchange adsorbents (PGMA-I, PGMA-II, PGMA-III) for the purification of glucoerucin from crude extracts of rocket seeds (*Eruca vesicaria*). Adsorbents that decolorized pigments and adsorbed/desorbed glucoerucin most efficiently were selected, and separation parameters optimized for purification efficiency.

## 2. Results

### 2.1. Adsorbent Performances of PGMA and Its Amine-Modified Derivatives

In this study, acrylic macroporous crosslinked copolymer PGMA beads were prepared: The mean particle diameter of the beads was about 150 μm; after sieving, the 100–150 μm fraction was used for the further reactions; the physicochemical properties of PGMA beads are shown in Table 1.

**Figure 1:**
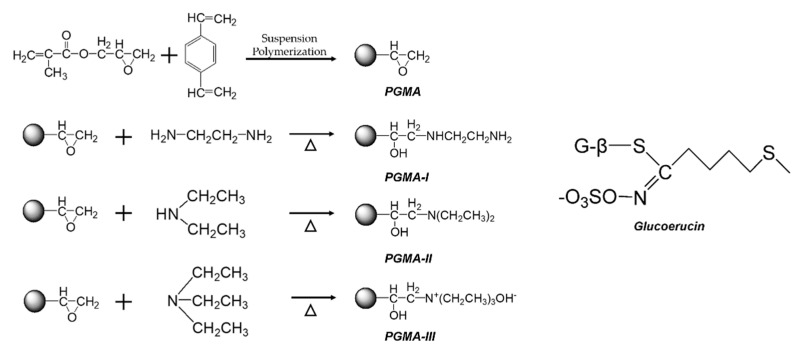
Synthetic route for PGMA, PGMA-I, PGMA-II and PGMA-III beads and the structure of glucoerucin.

Figure 1 shows the synthetic route for the preparation of PGMA, PGMA-I, PGMA-II and PGMA-III: The functional groups were covalently attached onto the surface of PGMA via reaction between the epoxy groups of the beads and amine groups of the amination reagents; the content of available epoxy groups on the bead surface was 2.31 mmol/g beads. The epoxide groups were a convenient means of immobilizing the amine functional groups: The O–C and N–C bonds formed were extremely stable; their contents in PGMA-I/PGMA-II/PGMA-III beads were 1.78/1.76/1.82 mmol/g, respectively.

**Figure 2:**
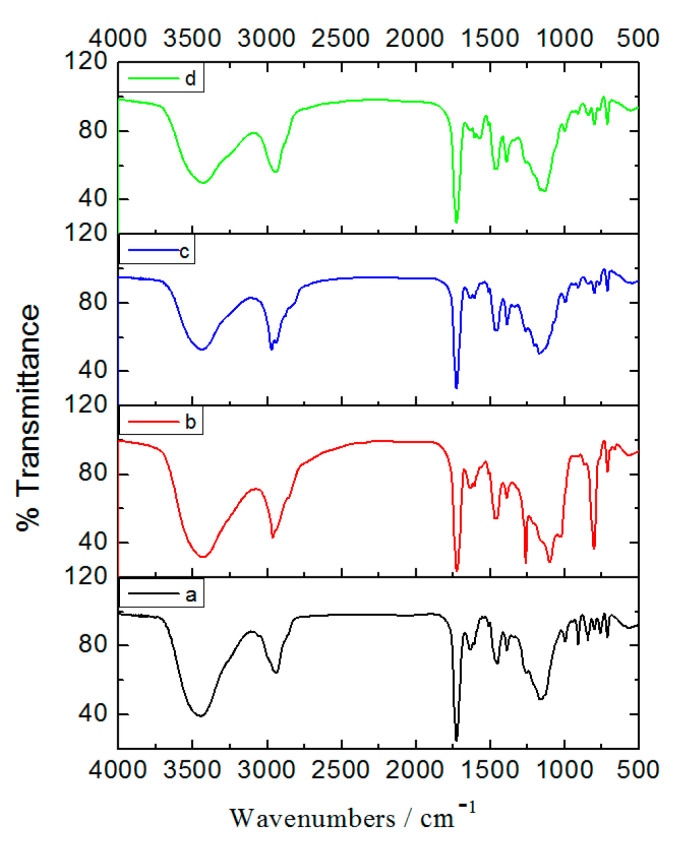
FT-IR spectra of prepared beads. (**a**) PGMA; (**b**) PGMA-I; (**c**) PGMA-II; (**d**) PGMA-III.

The Fourier transform infrared (FTIR) spectra of PGMA, PGMA-I, PGMA-II and PGMA-III beads are shown in Figure 2. The characteristic IR frequencies of PGMA and their assignments were: 1728 cm^−1^ (C=O stretch of GMA); 841 cm^−1^ and 907 cm^−1^ (epoxide ring deformation). Compared with PGMA, the peaks at 841 cm^−1^ and 907 cm^−1^ (epoxide ring deformation) were absent or much reduced for PGMA-I, PGMA-II and PGMA-III, consistent with the formation of the ethylenediamine/diethylamine/triethylamine derivatives.

**Figure 3:**
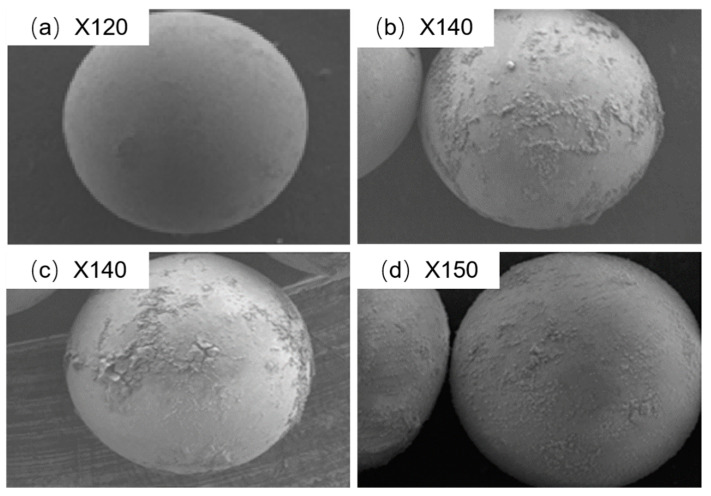
Morphology of prepared beads by SEM. (**a**) PGMA; (**b**) PGMA-I; (**c**) PGMA-II; (**d**) PGMA-III.

Figure 3 shows the morphology of the PGMA beads obtained using SEM. The uniform spherical shape of PGMA showed the formation of discrete particles on the surface layer (Figure 3b–d) after modification with each amine.

### 2.2. Static Adsorption and Desorption

**Figure 4:**
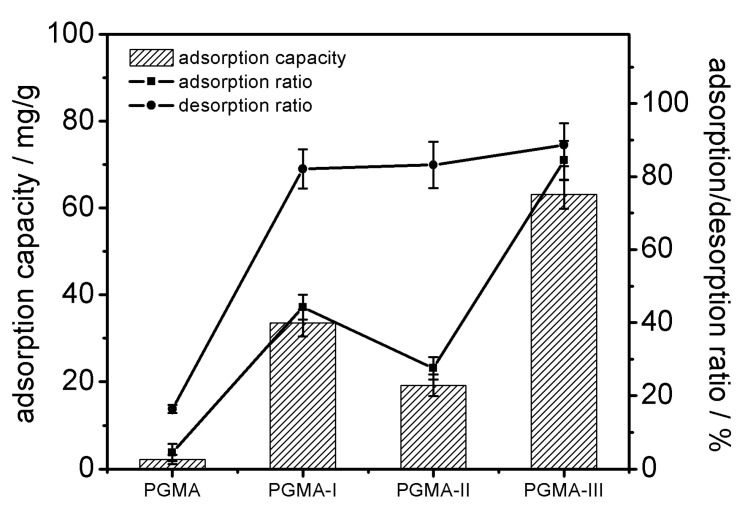
Adsorption capacities and the adsorption/desorption ratios for PGMA, PGMA-I PGMA-II and PGMA- III towards glucoerucin.

PGMA, PGMA-I, PGMA-II and PGMA-III were used to separate glucoerucin from crude extracts; the adsorption capacity and the ratios of adsorption and desorption of different adsorbents towards glucoerucin are shown in Figure 4. The adsorption/desorption ratio for PGMA-III towards glucoerucin was much higher than PGMA-I, PGMA-II and PGMA adsorbents. The high adsorption capacity of PGMA-III towards glucoerucin could be attributed to the strong interaction between the functional group -N^+^ (CH_3_)_3_ of PGMA-III and -SO_3_^−^ moiety of glucoerucin. Consequently, PGMA-III adsorbent was selected for further investigations of the adsorption/desorption behavior towards glucoerucin.

**Figure 5:**
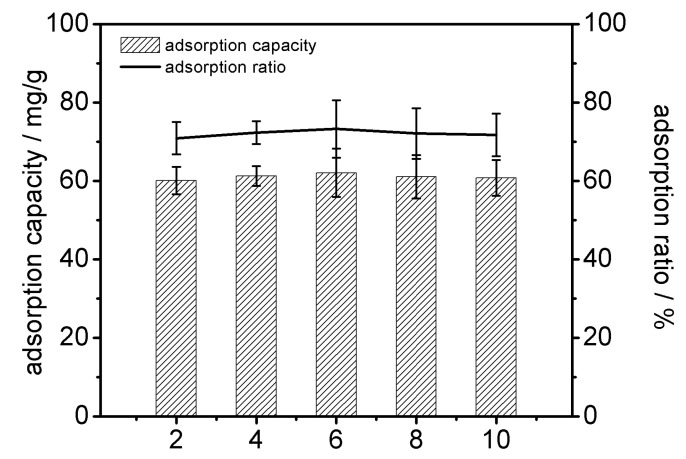
Effect of crude extracts solution pH on the adsorption capacity and adsorption ratios of PGMA-III adsorbent towards glucoerucin.

Figure 5 shows that the pH of crude extracts (pH range 2–10) had no significant effect on the adsorption capacities and adsorption/desorption ratios of PGMA-III. A high degree of ionic interaction still existed between the sulfate moiety -SO_3_^−^ of glucoerucin and the -N^+^(CH_3_)_3_ functional group of adsorbent which was not influenced by the change in pH; the ionization of the functional group of glucoerucin also played a major role in the adsorption process by PGMA-III. The pH of the crude extracts was adjusted to 6.2 for subsequent experiments.

The Langmuir equations and Freundlich equations are usually used to reveal the linearity fitting and interaction of solutes with the adsorbents [21,22,23].

The experimental data were fitted to the Langmuir equation:

(1) $$q_{e}=\frac{q_{0}KC_{e}}{1+KC_{e}}$$

where q is the adsorption capacity, K is the adsorption equilibrium constant (an empirical constant) and C_e_ is the concentration of adsorbate at equilibrium.

The experimental data were also fitted to the Freundlich equation:

(2) qe=KfCe1n

where K_f_ is the Freundlich constant (an indicator of adsorption capacity); and 1/n is an empirical constant related to the magnitude of the adsorption driving force [23].

Equilibrium adsorption isotherms for crude extracts of glucoerucin (40 mL) on PGMA-III (0.5 g) at different temperatures are summarized in Table 2. Although the correlation coefficients for the experimental fit of the adsorption of glucoerucin on PGMA-III were > 0.99 for both the Langmuir and Freundlich models, the Freundlich isotherm could provide a better prediction of the adsorption behavior. From the Freundlich equation, adsorption is likely to occur when 1/n lies between 0.1 and 0.5; it is unlikely when 1/n value lies 0.5 and 1; and its occurrence is least likely if 1/n value exceeds 1 [24]. Table 2 shows that the 1/n values were between 0.10 and 0.30, indicating that adsorption of glucoerucin on PGMA-III was favored. The results also demonstrated that the adsorption increased with increasing temperature. This suggested that a low temperature would inhibit adsorption and that the adsorption was endothermic.

**Figure 6:**
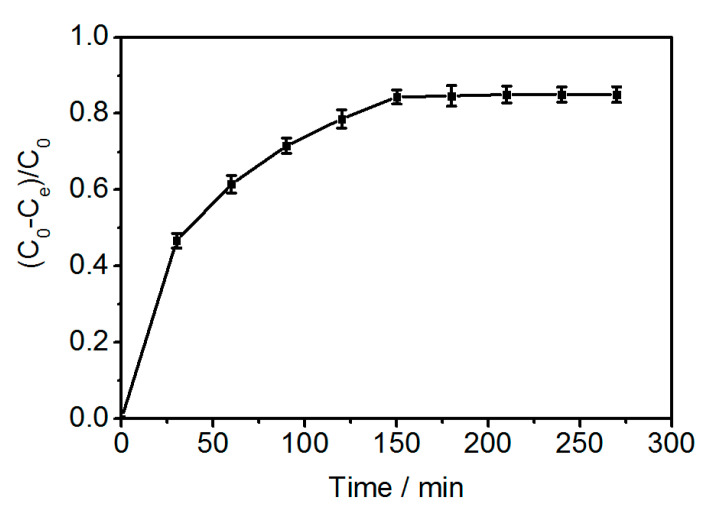
Adsorption kinetics for glucoerucin on PGMA-III adsorbent at 30 °C.

The kinetics of adsorption of glucoerucin on PGMA-III adsorbent at 30 °C are shown in Figure 6; the adsorption capacity increased with adsorption time to reach equilibrium after 150 min. The adsorption behavior may be consistent with the Freundlich multimolecular layer adsorption model arising from the strong interaction between -N^+^(CH_3_)_3_ of PGMA-III and -SO_3_^−^ moiety of glucoerucin. Two rate equations were used to determine the adsorption kinetics of glucoerucin on the PGMA-III adsorbent.

The pseudo-first-order rate equation of Lagergren is one of the most widely used for the adsorption of solute from a solution [25]. The model has the following form:

(3) $$\log^{q_{e}-q_{t}}=\log^{q_{e}}-k_{1}t_{}$$

where k_1_, q_e_ (mg/g), and q_t_ (mg/g) represent the rate constant of first-order adsorption (min^−1^), the amounts of adsorption at equilibrium and at time t (min), respectively.

The pseudo-second-order model [26] is expressed as:

(4) $$\frac{1}{q_{t}}=\frac{1}{k_{2}q_{e}^{2}t_{}}+\frac{1}{q_{e}}$$

where k_2_ represent the rate constant of pseudo-second-order adsorption (g/mg/min). The rate constant (k_2_) and adsorption at equilibrium (q_e_) can be obtained from the intercept and slope, respectively [27].

The parameters used for the pseudo-first-order and pseudo-second-order equations are shown in Table 3. The results showed that the theoretical q_e_ value estimated from the pseudo-second-order equation was close to the experimental value (R^2^ > 0.99).

**Figure 7:**
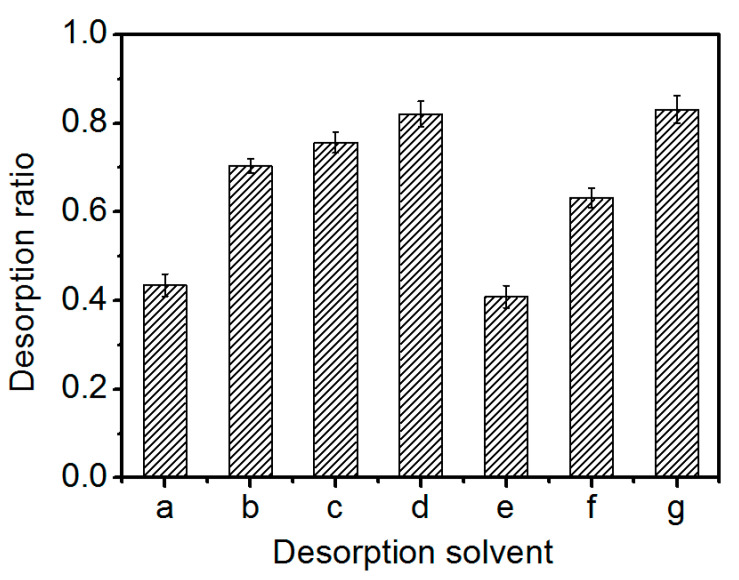
Effect of desorption solvent concentration on the static desorption ratio of glucoerucin from PGMA-III adsorbent at 30 °C. (**a**) 5% ammonia water solution; (**b**) 10% ammonia water solution; (**c**) 18% ammonia water solution; (**d**) 25% ammonia water solution; (**e**) 0.5 mol/L KCl solution; (**f**) 1 mol/L KCl solution; (**g**) 2 mol/L KCl solution.

The desorption solvent was selected according to the ionization of the adsorbent and the solubility of glucoerucin in the desorption solution. As shown in Figure 7, the desorption ratio of glucoerucin increased with increasing concentrations of aqueous ammonia or KCl. However, there were no significant differences between the maximum desorption ratios obtained using aqueous ammonia (82.08%) or KCl (83.12%) as the desorption solvents; either could be used to elute glucoerucin from the adsorbent.

### 2.3. Dynamic Adsorption and Desorption

When adsorption reaches the breakthrough point, the adsorption effect decreases and even ceases [28]. Hence, it is important to establish the breakthrough point to calculate the appropriate sample feed concentration and bed volume (BV) of the sample solution. The effect of feed concentration (0.657 mg/mL, 1.009 mg/mL, 1.502 mg/mL) at a flow rate of 2 BV/h (1 BV = 15 mL) on the adsorption capacity of PGMA-III was studied. As the feed concentration increased, the volume of adsorption solutions (eluate) at the 10% breakthrough point decreased; this also indicated that the process time decreased as the feed concentration increased. However, the adsorption capacity increased and reached its peak value (64.02 mg/g) at a feed concentration of 1.502 mg/mL. Therefore, based on the process time and the adsorption capacity, a feed concentration of 1.5 mg/mL and flow rate of 2 BV/h were selected for the following experiments.

**Figure 8:**
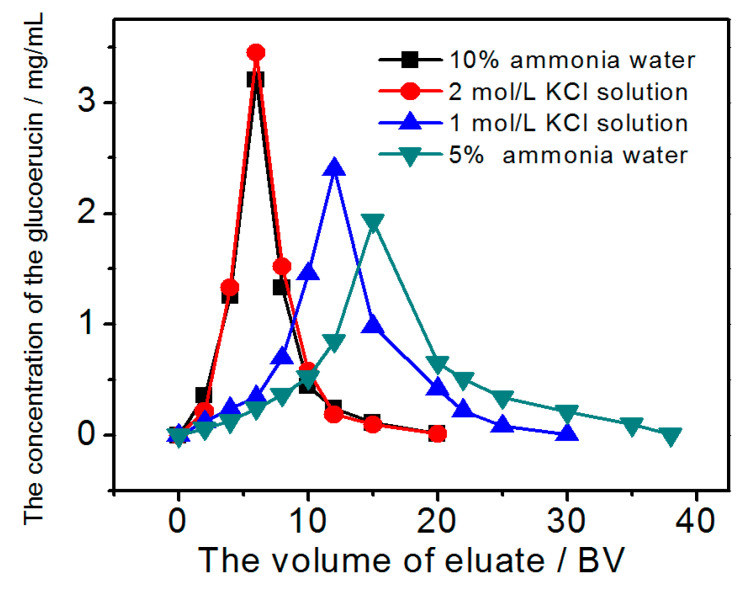
Effects of different concentrations of desorption solvents on the dynamic desorption of glucoerucin from PGMA-III adsorbent at 30 °C.

To optimize the elution of glucoerucin from PGMA-III, the effects of different concentrations of aqueous desorption solvents (ammonia 5%, 10%; KCl 1.0, 2.0 mol/L) were evaluated at a flow rate 2 BV/h and 30 °C. Figure 8 shows that the concentration glucoerucin increased in the eluate with increasing concentrations of aqueous ammonia or KCl. Maximum desorption occurred using 10% aqueous ammonia or 2.0 mol/L KCl solution, equivalent to desorption ratios of > 75% or > 82%, respectively; both dynamic desorption curves showed that glucoerucin eluted in a lower volume of eluate over a narrow range. Therefore, both 10% aqueous ammonia water and 2.0 mol/L KCl solution could be used as dynamic desorption solvents.

### 2.4. Decolorization Capacities of PGMA and Its Modified Adsorbents

A large amount of pigment remained in the eluate after dynamic desorption of glucoerucin using aqueous ammonia or KCl solution.

**Figure 9:**
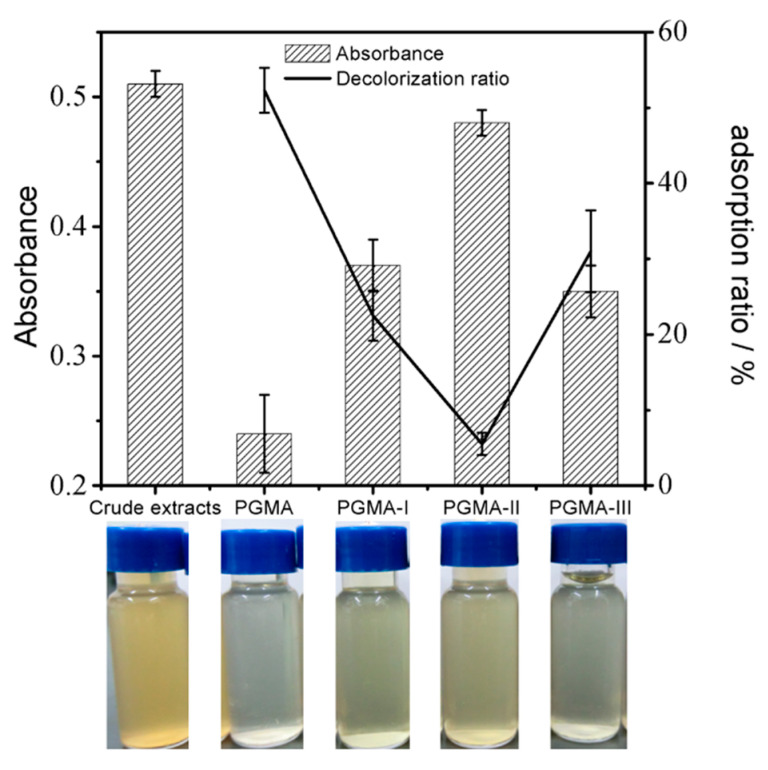
The effects of PGMA, PGMA-I, PGMA-II and PGMA-III on the UV absorbance and decolorization ratio of crude extracts with the visible color of the crude extracts and the crude extracts after treatment PGMA, PGMA-I, PGMA-II and PGMA-III.

Decolorization capacities of PGMA and its modified adsorbents were determined from the UV absorbance (420 nm) of crude extract solutions before and after decolorization [29]. As shown in Figure 9, the decolorization ratio of glucoerucin solution after treatment with PGMA adsorbent was much less than that after treatment with PGMA-I/PGMA-II/PGMA-III adsorbents; the color of the crude extracts after treatment with PGMA adsorbent showed greater clarity and transparency. Ethanol could be used as the regeneration solvent for PGMA adsorbent. This, and its minimal adsorption capacity towards glucoerucin (see Section 2.3) compared with the other adsorbents (PGMA-I, PGMA-II and PGMA-III), indicated that PGMA could be used effectively to decolorize crude extracts.

### 2.5. One Step Process of Decolorization and Separation

A one step process to decolorize and separate glucoerucin from crude extracts using two serially connected glass columns, wet-packed with selected adsorbents, was devised and tested: When the crude extracts flowed through the glass column containing PGMA, the pigments were absorbed by the particles; the decolorized crude extracts then flowed into the lower glass column where glucoerucin was adsorbed by PGMA-III. When the adsorption reached equilibrium, 10% aqueous ammonia (or 2 mol/L KCl solution) was used to elute glucoerucin; the collected eluate was evaporated and condensed into a solid product. After each cycle, 90% ethanol and 4% NaOH solution were applied respectively to regenerate the PGMA and PGMA-III adsorbents.

Table 4 shows the effects of the different elution solvents (10% aqueous ammonia; 2 mol/L KCl solution) on the purity and recovery of glucoerucin. When KCl solution was used as the desorption solvent, the purity of glucoerucin was low due to the presence of salt. Partial removal of KCl, by precipitation with methanol, increased the purity of glucoerucin by 48.8% while the recovery decreased by 35.07%. However, using 10% aqueous ammonia, the purity and recovery of glucoerucin increased from 60.52% to 74.39% and 55.48% to 80.63% respectively. Furthermore, consumption of electric (used for drying procedures) was significantly reduced and a large quantity of methanol was saved. Therefore, 10% aqueous ammonia could be effectively used to purify glucoerucin from crude extracts.

The purity of the glucoerucin obtained from the combined treatment of crude extracts with PGMA and PGMA-III was ≤74.39% higher than that only using PGMA-III alone; removal of the pigment with the PGMA increased the purity of glucoerucin (obtained using PGMA-III) by 11.30%. Therefore, PGMA and PGMA-III adsorbents could be used to decolorize and separate glucoerucin from other impurities using serially connected glass columns.

## 3. Materials and Methods

### 3.1. Materials

Glycidyl methacrylete (GMA) was purchased from J & K Technology Co., Ltd. (Beijing, China). Diethenyl benzene (DVB), ethylenediamine, diethylamine, triethylamine, HPLC-grade Methanol, and HPLC-grade trifluoroacetic acid (TFA) were purchased from Nankai University Chemical Factory (Tianjin, China). Other reagents were purchased from Beijing Chemical Factory (Beijing, China). Glucoerucin standard was purified from rocket seeds [10]. Rocket seeds were purchased from Institute of Vegetables and Flowers, China Academy of Agriculture Sciences (Beijing, China).

### 3.2. Preparation of Macroporous Crosslinked Copolymer and Its Amine Group-Modified Adsorbents

The method of oil-in-water thermal suspension polymerization was used to prepare the macroporous crosslinked copolymer with the methods reported previously [28]. Initially, 90 mL of deionized water was put into a 500 mL 3-mouth flask equipped with a mechanical stirrer, a thermometer, and a reflux condensation. In addition, then 0.5 g gelatin and 5 g sodium chloride were added as a dispersed phase into the 3-mouth flask under continuous agitation at 300 rpm for 0.5 h at 60 °C. Then, 10 mL GMA, 4 mL DVB, 7.5 mL toluene, 7.5 mL *n*-heptane were blended with 0.16 g BPO as a polymerization phase in a 100 mL beaker under ultrasound for 15 min at 60 °C. The polymerization phase was quickly added to the dispersed phase. The suspension was heated to 80 °C, 85 °C, 100 °C for 1 h, 2 h, 2.5 h under continuous stirring. After the reaction, the beads were washed with deionized water and ethanol. The product was dried in an under vacuum oven at 105 °C. The beads were used in further reactions. The macroporous crosslinked copolymer was nominated as PGMA.

The prepared 30 mL of PGMA beads was put into a 500 mL 3-mouth flask, and an equal volume of dioxane and deionized water was added to swell the beads. After that, 10 mL 0.1 mol/L of NaOH was added to the 500 mL 3-mouth flask, with a coupling agent of 90 mL ethylenediamine/diethylamine/triethylamine added into the above-mentioned system under continuous agitation at 200 rpm for 12 h at the set temperature 90 °C/50 °C/90 °C. The obtained beads were washed with hot water and reserved after being dried in an oven. The prepared anion-exchange beads were named as PGMA-Ⅰ (coupled with ethylenediamine), PGMA-Ⅱ (coupled with diethylamine) and PGMA-Ⅲ (coupled with diethylamine).

### 3.3. Characterization of Prepared Adsorbents

The size distribution and the mean diameter (dm) of the PGMA beads were determined by a laser particle size analyzer, Mastersizer 2000 (Malvern Instruments, Malvern, UK).

The main physicochemical properties of PGMA were measured as follows: water content (ω), shrinkage (Sr), wet density (ρp), porosity (P), pore volume (V), specific surface area (S) and mean pore diameter (D) of the matrices were measured with the methods reported previously [28].

The content of available epoxy groups in PGMA beads was determined using the pyridine–HCl method. The contents of available amino groups in PGMA beads modified by ethylenediamine/diethylamine/triethylamine were determined as follow: 0.2 g of the beads were allowed soak into water (10 mL) for 24 h. Then, 10 mL 2.0 mol/L HCl was added to the mixture and shaken for about 1.0 h. At the end of this period, the beads were filtered and assayed by titration with 2 mol/L NaOH solution [30].

FT-IR spectra of PGMA, PGMA-Ⅰ, PGMA-Ⅱ and PGMA-Ⅲ beads were obtained by using a FT-IR spectrophotometer, Nicolet170SX (Hitachi, Tokyo, Japan).

The morphology of the PGMA, PGMA-Ⅰ, PGMA-Ⅱ and PGMA-Ⅲ were observed by a scanning electron microscope (SEM), JEOL (Hitachi, Tokyo, Japan).

### 3.4. Preparation of Glucoerucin Crude Extracts

Rocket seeds were homogenized in a grinder. Then seed powder was put into boiled water and stirred for half an hour in a 10-fold excess (w/v). Then the clarified extract solution was obtained and centrifuged to remove solid residue. The glucoerucin extracts were subjected to quantitative analysis by HPLC.

### 3.5. HPLC Analysis of Glucoerucin

Quantification of glucoerucin concentration was carried out by a Shimadzu HPLC apparatus (Shimadzu, Kyoto, Japan), and a reversed-phase C18 column (250 mm × 4.6 mm,5 mm, Dianmonsil™, USA). The elution was made up of (A) methanol and (B) water with 0.1 % v/v TFA. The started elution was 1% A with 99% B, then the methanol was raised to 70% with 20 min. The flow rate was 1.0 mL/min. The column temperature and UV detector were set at 30 °C and 235 nm.

### 3.6. Static Adsorption and Desorption Experiments

The following equations were used to quantify the capacity of adsorption and adsorption/desorption ratio.

Capacity of adsorption:

(5) $$q_{e}=\frac{\left(C_{0}-C_{e}\right)\times V}{m}$$

Adsorption ratio:

(6) $$E=\frac{\left(C_{0}-C_{e}\right)}{C_{0}}\times 100\%\;$$

where q_e_ (mg/g) represents the adsorption capacity; E represents the adsorption ratio (%); C_0_ and C_e_ are the concentrations of glucoerucin in solution at the initial and equilibrium time, respectively (mg/mL), V and m represents the volume of the sample solution (mL) and the mass of the matrix (g).

Desorption ratio:

(7) $$D=\frac{C_{d}V_{d}}{\left(C_{0}-C_{e}\right)\times V}\times 100\%$$

where D represents the desorption ratio (%), C_d_ represents the concentration of glucoerucin in the eluent (mg/mL), V_d_ represents the volume of the eluent (mL), and C_0_, C_e_ and V are the same as defined above.

The static adsorption tests of crude extracts were carried out as follows: 0.5 g test adsorbent was put into a flask with a lid, 40 mL sample solution of glucoerucin extracts was added. The flask was then shaken at 150 rpm for 12 h at a constant temperature of 30 °C. The solutions before and after adsorption were analyzed by HPLC.

After the adsorption process reached equilibrium, the adsorbents were washed by deionized water. Then desorbed using a desorption solution which was ammonia water solution or KCl solution and shaking for 12 h at 30 °C.

The preliminary choice of adsorbent used to separate glucoerucin was evaluated by their capacities of adsorption and their ratios of adsorption and desorption. The adsorption and desorption properties were also compared, including the sample pH value and the concentration of ammonia water or KCl used for desorption. The adsorption isotherms of glucoerucin were studied. Their Langmuir and Freundlich equations were evaluated. In addition, adsorption kinetic curve was also evaluated by the pseudo-first-order kinetic model and pseudo-second-order kinetic model.

### 3.7. Dynamic Adsorption and Desorption Experiments

The adsorbent made by wet-packing was used in dynamic adsorption and dynamic desorption experiments. Glucoerucin solution was obtained through the column. After the adsorption process reached equilibrium, the column was washed with deionized water. Then it was eluted with desorption solvent. The effects of the concentration of feed and desorption solvent on the adsorption and desorption were studied. In addition, the concentration of glucoerucin was monitored by HPLC.

### 3.8. Decolorization Experiments

One gram of the tested adsorbents was mixed with 40 mL of sample solution of glucoerucin extracts and shake 4 h at 30 °C. The solutions were monitored by UV at 420 nm.

The following equation was used to quantify the ratio of decolorization.

Decolorization ratio:

(8) $$F=\frac{A_{0}-A_{}}{A_{0}}\times 100\%$$

where F is the decolorization ratio (%), A0 is the absorbance of crude extracts, and A is the absorbance of crude extracts after decolorization.

The adsorbent, with the highest decolorization ratio towards pigments and the lowest adsorption ratio to glucoerucin, would be selected out as the target adsorbent used to decolorize glucoerucin. In addition, the decolorization of the selected adsorbent was also analyzed by IR.

### 3.9. Experiments with Two Serially Connected Column 

A one step separation process was developed using two serially connected columns wet-packed with selected adsorbents to decolorize and separate glucoerucin from crud extracts. Two columns were serially connected. The first column, filled with the adsorbent, was used to decolorize the glucoerucin, and the second column, filled with another adsorbent, was used to separate and purify the glucoerucin. According to the above-mentioned process of adsorption and desorption experiments, the purity and the recovery of the product in different desorption solvents (ammonia water or KCl) used for desorption were evaluated. The purification effect before and after decolorization treatment were also studied.

### 3.10. Statistical Analysis

Data were expressed as mean ± SD of three duplicated experiments. Statistical analyses were performed using Super ANOVA v.1.11 software.

## 4. Conclusions

A simple, low-cost, and efficient method for the purification of GLs from cruciferous plants was described in our work. PGMA and amine-modified PGMA adsorbents (PGMA-I, PGMA-II and PGMA-III) were prepared and evaluated by comparing their adsorption/desorption and decolorization abilities for the purification of glucoerucin from crude extracts. According to the results, the strongly basic triethylamine modified PGMA (PGMA-III) adsorbent showed the best adsorption and desorption capacity of glucoerucin, and its adsorption data was a good fit to the Freundlich isotherm model and pseudo-second-order kinetics; the PGMA adsorbent gave the optimum decolorization performance. Furthermore, dynamic adsorption/desorption experiments were carried out to optimize the purification process. Two glass columns were serially connected and respectively wet-packed with PGMA and PGMA-III adsorbents so that glucoerucin could be decolorized and isolated from crude extracts in one process. Ammonia water and KCl solution were both used as the desorption solvent to separate glucoerucin. Compared with KCl solution, aqueous ammonia was a preferable desorption solvent for the purification of glucoerucin and overcame the challenges of desalination efficiency, residual methanol and high operation costs. The process of using serially connected glass columns, wet-packed with PGMA and PGMA-III, may provide a simple, low-cost, and efficient method for the purification of GLs from cruciferous plants.

## Figures and Tables

**Table 1:** Physicochemical properties of prepared PGMA beads.

Adsorbent	*ρ_p_* (g/mL)	*ω* (%)	*S_r_* (%)	*D* (nm)	*S* (m^2^/mL)	*V* (mL/g)	*P* (%)
PGMA	1.09	51.7	650	34.75	64.86	1.07	56.35

**Table 2:** Langmuir and Freundlich adsorption parameters of glucoerucin on PGMA-III adsorbent at different temperatures.

Temperature (°C)	Langmiur Model			Freundlich Model		
	q_o_ (mg/g)	K (mg/mL)	R^2^	K_f_ (mg/g)	1/n	R^2^
20	52.91	14.54	0.9916	52.69	0.1855	0.9924
30	64.94	7.333	0.9879	59.85	0.2737	0.9952
40	69.93	6.217	0.9880	63.28	0.2974	0.9893

**Table 3:** The pseudo-first-order kinetic model and pseudo-second-order kinetic model adsorption parameters of glucoerucin on PGMA-III adsorbent at 30 °C.

Experimental	Pseudo-First-Order Kinetic			Pseudo-Second-Order Kinetic		
q_exp_ (mg/g)	k_1_ (min^-1^)	q_e_ (mg/g)	R^2^	k_2_ (g/mg/min)	q_e_ (mg/g)	R^2^
60.24	0.0312	103.4	0.8803	0.0003922	71.43	0.9920

**Table 4:** Effects of desorption solvents on the purity and recovery of glucoerucin.

Desorption Solvent	Purity (%)	Recovery (%)
2 mol/L KCl	40.66	85.45
2 mol/L KCl + desaltation *	60.52	55.48
10% ammonia water	74.39	80.63

* Precipitation with methanol to remove salt from GLs.

## References

[1] Blažević I., Montaut S., Burčul F., Olsen C.E., Burow M., Rollin P., Agerbirk N. (2020). Glucosinolate structural diversity, identification, chemical synthesis and metabolism in plants. Phytochemistry.

[2] Bones A.M., Rossiter J.T. (2006). The enzymic and chemically induced decomposition of glucosinolates. Phytochemistry.

[3] Dayalan Naidu S., Suzuki T., Yamamoto M., Fahey J.W., Dinkova-Kostova A.T. (2018). Phenethyl isothiocyanate, a dual activator of transcription factors NRF2 and HSF1. Mol. Nutr. Food Res..

[4] Gillespie S., Holloway P.M., Becker F., Rauzi F., A Vital S., Taylor K., Stokes K.Y., Emerson M., Gavins F.N. (2018). The isothiocyanate sulforaphane modulates platelet function and protects against cerebral thrombotic dysfunction. Brit. J. Pharmacol..

[5] Jaja-Chimedza A., Zhang L., Wolff K., Graf B.L., Kühn P., Moskal K., Carmouche R., Newman S., Salbaum J.M., Raskin I. (2018). A dietary isothiocyanate-enriched moringa (*Moringa oleifera*) seed extract improves glucose tolerance in a high-fat-diet mouse model and modulates the gut microbiome. J. Funct. Foods.

[6] Zhang Y., Talalay P., Cho C.G., Posner G.H. (1992). A major inducer of anticarcinogenic protective enzymes from broccoli: Isolation and elucidation of structure. Proc. Natl. Acad. Sci. USA.

[7] Dinkova-Kostova A.T., Fahey J.W., Kostov R.V., Kensler T.W. (2017). KEAP1 and done? Targeting the NRF2 pathway with sulforaphane. Trends Food Sci. Tech..

[8] Hanlon N., Coldham N., Sauer M.J., Ioannides C. (2009). Modulation of rat pulmonary carcinogen-metabolising enzyme systems by the isothiocyanates erucin and sulforaphane. Chem-Biol. Interact..

[9] Greaney A.J., Maier N.K., Leppla S.H., Moayeri M. (2016). Sulforaphane inhibits multiple inflammasomes through an Nrf2-independent mechanism. J. Leukoc. Biol..

[10] Barillari J., Canistro D., Paolini M., Ferroni F., Pedulli G.F., Iori R., Valgimigli L. (2005). Direct antioxidant activity of purified glucoerucin, the dietary secondary metabolite contained in rocket (*Eruca sativa* Mill.) seeds and sprouts. J. Agric. Food Chem..

[11] Charpentier N., Bostyn S., Coïc J.P. (1998). Isolation of a rich glucosinolate fraction by liquid chromatography from an aqueous extract obtained by leaching dehulled rapeseed meal (*Brassica napus* L.). Ind. Crop. Prod..

[12] Kuang P., Liang H., Yuan Q. (2010). Isolation and purification of glucoraphenin from radish seeds by low-pressure column chromatography and nanofiltration. Sep. Sci. Technol..

[13] Rochfort S., Caridi D., Stinton M., Trenerry V.C., Jones R. (2006). The isolation and purification of glucoraphanin from broccoli seeds by solid phase extraction and preparative high performance liquid chromatography. J. Chromatogr. A.

[14] Fahey J.W., Wade K.L., Stephenson K.K., Chou F.E. (2003). Separation and purification of glucosinolates from crude plant homogenates by high-speed counter-current chromatography. J. Chromatogr. A.

[15] Visentin M., Tava A., Iori R., Palmieri S. (1992). Isolation and identification for trans-4-(methylthio)-3-butenyl glucosinolate from radish roots (*Raphanus sativus* L.). J. Agric. Food Chem..

[16] Fahey J.W., Wade K.L., Stephenson K.K., Panjwani A.A., Liu H., Cornblatt G., Cornblatt B.S., Ownby S.L., Fuchs E., Holtzclaw W.D. (2019). Bioavailability of sulforaphane following ingestion of glucoraphanin-rich broccoli sprout and seed extracts with active myrosinase: A pilot study of the effects of proton pump inhibitor administration. Nutrients.

[17] Toribio A., Nuzillard J.M., Renault J.H. (2007). Strong ion-exchange centrifugal partition chromatography as an efficient method for the large-scale purification of glucosinolates. J. Chromatogr. A.

[18] Du Q., Fang J., Gao S., Zeng Q., Mo C. (2008). A gram-scale separation of glucosinolates from an oil-pressed residue of rapeseeds using slow rotary countercurrent chromatography. Sep. Purif. Technol..

[19] Wang T., Liang H., Yuan Q. (2012). Separation of sinigrin from Indian mustard (*Brassica juncea* L.) seed using macroporous ion-exchange resin. Korean J. Chem. Eng..

[20] Li J., Chase H.A. (2010). Development of adsorptive (non-ionic) macroporous resins and their uses in the purification of pharmacologically-active natural products from plant sources. Nat. Prod. Rep..

[21] Gamoudi S., Srasra E. (2019). Adsorption of organic dyes by HDPy+-modified clay: Effect of molecular structure on the adsorption. J. Mol. Struct..

[22] Carmo A.M., Hundal L.S., Thompson M.L. (2000). Sorption of hydrophobic organic compounds by soil materials: Application of unit equivalent Freundlich coefficients. Environ. Sci. Technol..

[23] Wu X., Liu Y., Liu Y., Di D. (2015). Evaluation on the adsorption capability of chemically modified macroporous adsorption resin with ionic liquid. Colloid. Surf. A.

[24] Li R., Zhao R., Zhang H., Li C., Feng D., Qin P., Tan T. (2010). A Novel Medium Poly (vinyl acetate-triallyl isocyanurate-divinylbenzene) Coupled with Oligo-β-Cyclodextrin for the Isolation of Puerarin from Pueraria Flavones. Chromatographia.

[25] Zou X., Pan J., Ou H., Wang X., Guan W., Li C., Yan Y., Duan Y. (2011). Adsorptive removal of Cr (III) and Fe (III) from aqueous solution by chitosan/attapulgite composites: Equilibrium, thermodynamics and kinetics. Chem. Eng. J..

[26] Ho Y.S., McKay G. (1999). Pseudo-second order model for sorption processes. Process. Biochem..

[27] Rudzinski W., Plazinski W. (2006). Kinetics of solute adsorption at solid/solution interfaces: A theoretical development of the empirical pseudo-first and pseudo-second order kinetic rate equations, based on applying the statistical rate theory of interfacial transport. J. Phys. Chem. B.

[28] Song H.B., Xiao Z.F., Yuan Q.P. (2009). Preparation and characterization of poly glycidyl methacrylete–zirconium dioxide–β-cyclodextrin composite matrix for separation of isoflavones through expanded bed adsorption. J. Chromatogr. A.

[29] Yang W., Shi X., Wang J., Chen W., Zhang L., Zhang W., Zhang X., Lu J. (2019). Fabrication of a novel bifunctional nanocomposite with improved selectivity for simultaneous nitrate and phosphate removal from water. ACS Appl. Mater. Inter..

[30] Bayramoğlu G., Arıca M.Y. (2005). Ethylenediamine grafted poly (glycidylmethacrylate-co-methylmethacrylate) adsorbent for removal of chromate anions. Sep. Purif. Technol..

